# Graphene Oxide and Biomolecules for the Production of Functional 3D Graphene-Based Materials

**DOI:** 10.3389/fmolb.2022.774097

**Published:** 2022-03-15

**Authors:** Paolo Passaretti

**Affiliations:** ^1^ Institute of Cancer and Genomic Sciences, School of Medical and Dental Sciences, University of Birmingham, Birmingham, United Kingdom; ^2^ School of Chemical Engineering, University of Birmingham, Birmingham, United Kingdom

**Keywords:** graphene oxide, biomolecules, DNA, proteins, enzymes, self-assembly, 3D functional materials

## Abstract

Graphene and its derivatives have been widely employed in the manufacturing of novel composite nanomaterials which find applications across the fields of physics, chemistry, engineering and medicine. There are many techniques and strategies employed for the production, functionalization, and assembly of graphene with other organic and inorganic components. These are characterized by advantages and disadvantages related to the nature of the specific components involved. Among many, biomolecules and biopolymers have been extensively studied and employed during the last decade as building blocks, leading to the realization of graphene-based biomaterials owning unique properties and functionalities. In particular, biomolecules like nucleic acids, proteins and enzymes, as well as viruses, are of particular interest due to their natural ability to self-assemble *via* non-covalent interactions forming extremely complex and dynamic functional structures. The capability of proteins and nucleic acids to bind specific targets with very high selectivity or the ability of enzymes to catalyse specific reactions, make these biomolecules the perfect candidates to be combined with graphenes, and in particular graphene oxide, to create novel 3D nanostructured functional biomaterials. Furthermore, besides the ease of interaction between graphene oxide and biomolecules, the latter can be produced in bulk, favouring the scalability of the resulting nanostructured composite materials. Moreover, due to the presence of biological components, graphene oxide-based biomaterials are more environmentally friendly and can be manufactured more sustainably compared to other graphene-based materials assembled with synthetic and inorganic components. This review aims to provide an overview of the state of the art of 3D graphene-based materials assembled using graphene oxide and biomolecules, for the fabrication of novel functional and scalable materials and devices.

## Introduction

Since 1986, when the term “graphene” was proposed to indicate the two-dimensional form of crystalline carbon (Boehm et al., 1986), a variety of graphene derivatives have been classified over time based on their specific chemical bonds between carbons. Currently, this classification includes diamond, graphite, fullerene and carbyne families (Inagaki and Kang, 2014). Graphene is defined as a single layer of *sp* ^*2*^-hybridized carbon atoms and it continuously triggers the interest of the scientific communities since its first characterization in 2004 (Novoselov et al., 2004). It is a 2D allotrope of carbon in the form of sheets with the thickness of an atom, characterized by a honeycomb network π-conjugated which shows a variety of physical and chemical properties (Yang et al., 2013). Its intrinsic properties are also appreciated for the ability to effectively work and enhance other nanomaterials performances when combined. This is currently leading to the production of novel functional nanostructures and nanocomposites with advanced performances for many applications across several scientific fields (Allen et al., 2010; Krishna et al., 2013; Chabot et al., 2014; Kravets et al., 2015; Shehzad et al., 2016; Ahmad et al., 2018; Mohan et al., 2018; Maleki et al., 2020). Graphene shows unique properties including, a half-integer quantum Hall effect for both electrons and holes at room temperature (Novoselov et al., 2005; Zhang et al., 2005; Novoselov et al., 2006; Novoselov et al., 2007; Georgakilas et al., 2012), extraordinary high carrier mobility, and single-molecule detection (Schedin et al., 2007). Graphene also exhibits other remarkable electronic, mechanical and optical characteristics. These include ambipolar field-effect (Lee et al., 2011), high mechanical strength (Byun, 2015), large specific surface area (Elías et al., 2010), high-transparency (Chang et al., 2011; Zhao et al., 2014) and excellent electric and thermal conductivity (Eda and Chhowalla, 2010). Due to its extraordinary and superior properties, graphene has already demonstrated its potential in a broad range of applications such as the manufacturing of capacitors, fuel cells, batteries, sensors, transparent conductive films, high-frequency circuits, absorbers and flexible electronics (Sutter et al., 2008; Chang et al., 2011; Mattevi et al., 2011; Bitounis et al., 2013; Pinto et al., 2013; Nezakati et al., 2014).

Despite its great potential for many applications, it is generally difficult to scale up the properties of individual graphene nanosheets to macroscopic materials. Moreover, considering that graphene shows zero band gap as well as inertness to many chemical reactions, it is less competitive than other materials for the fabrication of semiconductors and sensors. To overcome these limitations, there is a considerable increase in the number of research studies aiming to improve the functionalization of graphene and its derivatives (Chen et al., 2012; Bottari et al., 2017; Yu et al., 2020). Graphenes such as graphene, graphene oxide (GO) and reduced graphene oxide (rGO), can be functionalized with a wide range of molecules, polymers and nanoparticles *via* covalent bonds and non-covalent interactions (Georgakilas et al., 2012). In particular, GO due to the presence of chemical groups containing oxygen, shows a superior dispersibility in aqueous-based buffers than other more hydrophobic graphenes (Georgitsopoulou et al., 2021). Therefore, GO is considered a better candidate for bio-functionalization. Also, its relatively lower toxicity makes GO a better choice for biomedical applications (Goenka et al., 2014; Nezakati et al., 2014). Another advantage of using GO to produce graphene-based materials (GBMs) is that GO can naturally assemble with biomolecules in aqueous solutions. Moreover, it can be subsequently reduced to rGO *via* removing the oxygen-containing groups, partially restoring the properties of pristine graphene (Ardini et al., 2016; Mohan et al., 2018).

Among the wide range of functionalizations, the bio-functionalization of graphenes with nucleic acids, peptides, proteins, enzymes and entire virus particles (e.g., M13 bacteriophage (Passaretti et al., 2019)) for the production of GBMs, is particularly appealing. This is leading to novel fields in biotechnology and synthetic biology (Goenka et al., 2014; Li et al., 2016; Wang et al., 2017). In particular, bio-functionalization is useful to design and produce composites with a *bottom-up* approach combining the components through self-assembly strategies. Bio-components are characterized by high-specificity of binding as well as the possibility to work at standard conditions of temperature, pressure and pH. Among the numerous advantages given by the bio-components and the bio-functionalization, it is also important to highlight the reduction of costs, scalability and environmental sustainability, without affecting the performances and quality of materials (Georgakilas et al., 2012; Hu et al., 2015).

This review aims to provide an overview of the different graphene derivatives and their production methods. The interactions leading to the assembly and possible functionalizations of graphene-based materials (GBMs) is discussed, giving particular attention to the class of 3D graphene oxide-based biomaterials (GOBBs). In particular, examples of electrospun fibres, cast films, freeze-dried sponges and 3D printed custom shaped materials are described. Moreover, although this review is focused on a specific group of biomolecules (e.g., nucleic acids, proteins and enzymes), other biopolymers are also discussed due to their importance and substantial employment in combination with GO, especially for the production of fibres and 3D printed GOBBs.

## Graphene, Graphene Oxide and Reduced Graphene Oxide

**FIGURE 1:**
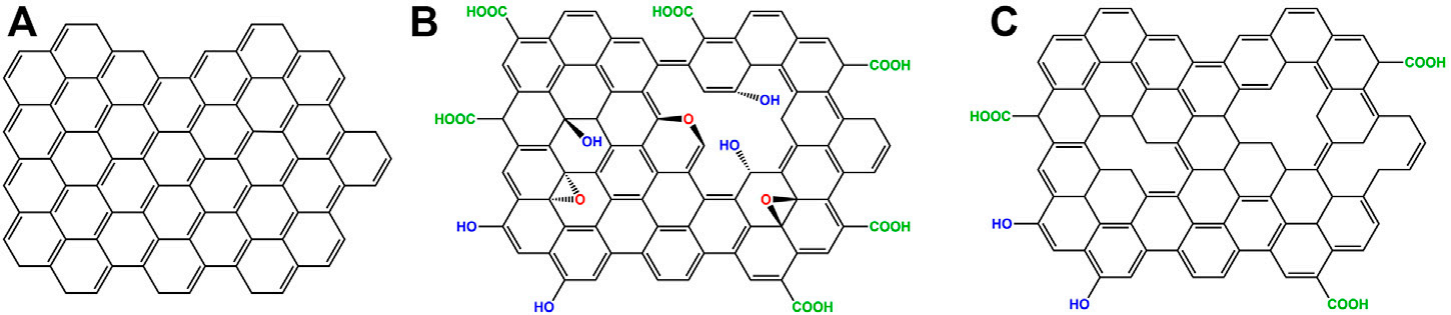
Graphene, GO and rGO structures **(A)** Graphene is a single layer of carbon atoms arranged in a honeycomb lattice. **(B)** Graphene oxide (GO), has hydrophilic groups containing oxygen protruding outside its plane and edges. **(C)** Reduced graphene oxide (rGO) has fewer hydrophilic groups compared to GO, due to a reduction process. Contrarily to pristine graphene, the carbon atoms of GO and rGO do not lie in the same plane due to the presence of structural defects.

Individual graphene nanosheets were isolated for the first time by simply peeling a piece of graphite with scotch tape through a process called mechanical exfoliation by Andre Geim and Konstantin Novoselov at the University of Manchester. Due to this extraordinary study published in 2004 (Novoselov et al., 2004), they were awarded the Nobel Prize in Physics in 2010. The resulting graphite monolayer, known as graphene **(** Figure 1A **)**, is now considered the thinnest stable material known to exist (<1 nm) and it completely revolutionized research across many scientific fields (Allen et al., 2010; Krishna et al., 2013; Chabot et al., 2014; Kravets et al., 2015; Shehzad et al., 2016; Ahmad et al., 2018; Mohan et al., 2018; Geetha Bai et al., 2019; Dhinakaran et al., 2020; Maleki et al., 2020). Graphene has the thickness of a carbon atom characterized by extraordinary mechanical, electrical and thermal properties such as fracture toughness 4.0 MPa m^0.5^ (Zhang et al., 2014), Young’s modulus 1.0 TPa (Lee et al., 2008), electronic mobility 15,000–200,000 cm^2^ V^−1^ s^−1^ (Bolotin et al., 2008) and thermal conductivity 4.84–5.30 × 10^3^ W m^−1^ K^−1^ (Balandin et al., 2008). Therefore, a large number of heterostructures and composites have been produced by combining graphene sheets with other components (Georgakilas et al., 2012; Bottari et al., 2017; Mohan et al., 2018; Ma et al., 2019; Yu et al., 2020).

Another appealing derivative of graphene is GO (Figure 1B), which is a 2D nanomaterial obtained from the oxidation of graphite. GO has good dispersibility in aqueous buffers, due to the presence of oxygen-containing groups such as epoxides, carboxyls, and hydroxyls distributed along the edges and on its planar surface, which provide electrostatic repulsion between the sheets. Moreover, it has been shown that these oxygen-containing groups facilitate the functionalization of GO with other molecules and the fabrication of GBMs employed for drug-delivery, bioanalysis and other biomedical applications (Sun et al., 2008; Zhang et al., 2010; Liu et al., 2013; Goenka et al., 2014). There are numerous studies conducted in several cell lines about the cytotoxicity of graphenes where GO often shows the best biocompatibility (Lu et al., 2009; Hu et al., 2011; Liao et al., 2011; Wang et al., 2011; Zhang et al., 2012). Commonly, cytotoxicity of graphenes is associated with final cell viability lower than 80% (Coleman et al., 2017). However, both the definition of cytotoxicity for graphene and its actual biocompatibility is currently a controversial topic in the field of GBMs which needs to be further investigated and discussed (Coleman et al., 2017).

Although GO cannot compete with pristine graphene in terms of stiffness and electron mobility, the oxygen-containing groups facilitate the interaction with bio-components especially *via* several weak intermolecular interactions (Georgakilas et al., 2012; Li et al., 2016). Many reduction procedures have been developed to convert GO into graphene. These procedures aim to remove the oxygen-containing groups and structural defects to recover the π-conjugate honeycomb of graphene, therefore, restoring its properties (Mohan et al., 2018). Although numerous efforts have been made, the reduction methods developed so far have not reached the set expectations (Kumar et al., 2021). Residual functional groups and other defects drastically alter the structure of the carbon plane and it is still not appropriate to refer to the rGO (Figure 1C), simply as if it was graphene, given that their properties are substantially different (Table 1) (Mohan et al., 2018; Kumar et al., 2021).

**TABLE 1:** Graphenes characteristics.

Property	Graphene	Ref	GO	Ref	rGO	Ref
Area density	0.3818 Å^−2^	Zhao et al. (2013)	N/A	—	N/A	—
Breaking strength	42 N m^−1^	Lee et al. (2008)	N/A	—	N/A	—
Cohesive energy	291 mJ m^−2^	Zhao et al. (2013)	N/A	—	N/A	—
Interlayer equilibrium spacing	3.41 Å	Zhao et al. (2013)	N/A	—	N/A	—
Electronic mobility	200 × 10^3^ cm^2^ V^−1^ s^−1^ at electron densities of ∼2 × 10^11^ cm^−2^	Bolotin et al. (2008)	N/A	—	2–200 cm^2^ V^−1^ s^−1^	Gómez-Navarro et al. (2007)
Fracture toughness	4.0 ± 0.6 MPa m^0.5^	Zhang et al. (2014)	N/A	—	N/A	—
Intrinsic strength	130 GPa	Lee et al. (2008)	76–293 MPa	Gao et al. (2011)	N/A	—
Magnetism	Strongly diamagnetic	Sepioni et al. (2010)	Weak superparamagnetic	Sarkar et al. (2014)	Weak superparamagnetic	Sarkar et al. (2014)
Melting point	4,510 K	Los, (2015)	N/A	—	N/A	—
Opacity	2.3%	Nair et al. (2008)	N/A	—	N/A	—
		Zhu et al. (2010)				
Optical transmittance	97.7%	Nair et al. (2008)	N/A	—	70–80%	(Becerril et al., 2008;
		Zhu et al. (2010)				Wang et al., 2008)
Second- and third-order elastic stiffness	340 ± 50 N m^−1^ and -690 ± 120 N m^−1^	Lee et al. (2008)	N/A		N/A	
Sheet resistance	*R*_*SK*_ 670 Ω/sq	Peng et al. (2015)	276–2024 Ω/sq	Kang et al. (2013)	N/A	—
	*R*_*SH*_ 1840 Ω/sq					
Solubility in water	Non-soluble	—	6.6 μg ml^−1^	Konios et al. (2014)	4.74 μg ml^−1^	Konios et al. (2014)
Specific surface area	Theoretical	Zhu et al. (2010)	Theoretical	Zhang et al. (2020)	N/A	—
			2,418 m^2^ g^−1^			
			Measured			
			2,391 m^2^ g^−1^			
Thermal conductivity	4.84–5.30 × 10^3^	Balandin et al. (2008)	72–670 W m^−1^ K^−1^	Chen and Li, (2020)	N/A	—
	W m^−1^ K^−1^					
Young’s modulus	1.0 ± 0.1 TPa	Lee et al. (2008)	Ordered GO	Liu et al. (2012)	N/A	—
			380–470 GPa			
			Amorphous GO			
			290–430 GPa			

*These values are highly variable due to the specific amount of oxygen-containing groups and structural defects.

### Production Methods

Most of the research studies do not refer to pristine graphene due to the limited preparation yield (Mohan et al., 2018). Moreover, graphene derivatives such as GO and rGO are more commonly available and characterized by similar properties compared to graphene. Although most researchers call these materials “graphene,” it is misleading to identify them as such, given their heteroatomic irregularity, impurity and structural defects. Therefore, their nomenclature is expected to be corrected and standardised more accurately in the future. There are two main approaches to synthesising graphenes which can be classified as top-down and bottom-up depending on the starting materials employed.

#### Top-Down Methods

Although mechanical exfoliation can directly produce high-quality graphene, it is not suitable for large-scale production (Kumar et al., 2021). To overcome the limitation of this method, other top-down approaches to produce graphene in bulk have been developed such as liquid-phase exfoliation (LPE) (Hernandez et al., 2008), electrochemical exfoliation (Achee et al., 2018), and chemical oxidation-reduction (Marcano et al., 2010; Polsen et al., 2015; Kumar et al., 2021). The latter is the preferred method among the top-down approaches since it does not require particularly sophisticated equipment and produces large sheets of GO characterized by a high density of functional groups, which facilitate the introduction of further functionalizations (Park and Ruoff, 2009). This method is also known as the oxidation-exfoliation-reduction process and begins with the chemical oxidation of graphite to graphite oxide, followed by its exfoliation to produce single GO layers, which are finally reduced to rGO with a higher carbon-oxygen ratio than GO (Bai et al., 2011). The GO precursor is graphite oxide which exposes functional groups containing oxygen and retains a structure similar to that of stacked graphite (Benzait et al., 2021). The history of the preparation of graphite oxide can be traced back to 1859 when steaming KClO_3_ and HNO_3_ were used to oxidize graphite powder, leading to the formation of a material with a carbon-oxygen ratio of about 2:1 (Brodie, 1859). KClO_3_ and HNO_3_ must be handled with particular caution due to the generation of highly toxic ClO_2_ gas and the possibility of triggering an explosive reaction (Compton and Nguyen, 2010). Hummers and Offeman, in 1958 developed an approach to synthesise GO with a similar level of oxidation but using different reagents (concentrated NaNO_3_, KMnO_4_, and H_2_SO_4_) (Hummers and Offeman, 1958). Using the Hummers’ method or its variants, GO can be produced in a relatively short time (1–24 h) (Ikram et al., 2020a), as well as avoid the generation of ClO_2_ by using a more recent modified version of the protocol (Marcano et al., 2010; Yu et al., 2016). The exfoliation of the graphite oxide in a single layer GO can be obtained by using additional energy inputs, such as mechanical agitation or ultrasound (Li et al., 2008). The latter is a more effective method for reducing the size and separating stacked GO sheets (Pan and Aksay, 2011). Several parameters, including the size of starting material, oxidation procedures and forms of energy for exfoliation, play an important role in controlling the size or number of layers of GO. By increasing the oxidation or the sonication time, or the number of oxidants, the average size of the GO flakes can be adjusted between 550 and 59,000 nm^2^ (Zhang et al., 2009; Coleman et al., 2017). The number of GO layers in the final products is strongly dependent on the size and crystallinity of the graphite precursors (Wu et al., 2009). The oxidation of graphite using strong oxidizing agents (e.g., KClO_3_, HNO_3_, KMnO_4_ and K_2_FeO_4_) leads to the breakage of the π-bonds and therefore, the conductivity of the oxidized products is drastically reduced. To remove the functional groups containing oxygen it is necessary to perform reduction processes which partially improve the electrical conductivity by restoring the π-conjugated network (Chen et al., 2012). Therefore, several reduction strategies have been developed so far, including chemical, electrochemical, thermal, hydrothermal or solvothermal, UV light and microbial reduction (Gao and Duan, 2015; Kumar et al., 2021). However, none of these methods is currently efficient enough to convert GO into high-quality graphene. Apart from the thermal reduction, not much progress has been made in the synthetic methodologies used to reduce GO into rGO since Boehm *et al.* (Boehm et al., 1962; Dreyer et al., 2010a; Dreyer et al., 2010b). Moreover, to reduce the use of polluting agents employed in the oxidation-exfoliation-reduction process, alternative eco-friendly methods to reduce GO using plant extracts (Jha et al., 2021; Meka Chufa et al., 2021) or industrial waste precursors have been recently tested (Ikram et al., 2020b).

#### Bottom-Up Methods

Alternatively to the production methodologies described above, there are the bottom-up methods that are based on the use of hydrocarbons compounds as precursors (Lim et al., 2018; Kumar et al., 2021). These methods include epitaxial growth (Sutter et al., 2008), thermal pyrolysis (Amirov et al., 2015), laser-assisted synthesis (Bobb et al., 2020), organic synthesis (Wu et al., 2007) and chemical vapour deposition (CVD) (Mattevi et al., 2011; Saeed et al., 2020). The latter was firstly described in 2006 by Somani et al. and is one of the most popular bottom-up approaches due to the relatively simple procedure and reaction control (Somani et al., 2006). The process consists in injecting CH_4_, H_2_ and Ar into a horizontal quartz tube of about 1 m in length and 5 cm in diameter. Inside the quartz tube which serves as a reactor chamber, there is a substrate with a conductive metal such as Ni or Cu, onto which multi- or mono-layered graphene will form at a temperature comprised between 200 and 1,000°C (Li et al., 2011). Although the CVD method and its variants are scalable, it requires sophisticated instruments, a considerable amount of energy and time, which make this method particularly expensive compared to others (Beloin-Saint-Pierre and Hischier, 2021).

In summary, there are numerous top-down and bottom-up methods to synthesise graphene on a large scale. However, the methods characterized by larger yields, shorter production time and lower costs, usually provide lower quality graphene and also employ polluting reagents. On the other hand, the methods that can synthesise high-quality graphene are limited by the long production time and high costs involved for the equipment needed. Therefore, the optimization of graphene production methods or the design of novel and more sustainable approaches is crucial for the advancement of the graphene-related scientific fields as well as for the development and commercialization of novel functional GBMs (Beloin-Saint-Pierre and Hischier, 2021).

## Graphene-Based Materials

Graphene and its derivatives have given an incredible boost to the development of alternative carbon-based materials. The 2D structure of graphene, GO and rGO makes them very appealing components employable as versatile building blocks for the fabrication of nanostructured materials and devices (Shehzad et al., 2016; Bottari et al., 2017; Mohan et al., 2018). Besides pristine graphene, GO and rGO, there are other zero and one dimensional (0D and 1D) carbon-based nanomaterials such as fullerenes, graphene quantum dots (GQDs), nanodiamonds, graphene nanoribbons (GNRs), single and multi-walled carbon nanotubes (CNTs) (Inagaki and Kang, 2014). These derivatives show similar chemical and physical properties, but different structural characteristics and can be employed as alternative building blocks (Shehzad et al., 2016; Bottari et al., 2017; Ma et al., 2019). Graphenes have been widely employed as the main constituent of novel composite materials with enhanced and new properties. Graphenes are promising components in the development of fluorescent biosensors, due to their quenching capability toward various organic dyes and quantum dots (Chen et al., 2010; Yi et al., 2011a; Yi et al., 2011b) as well as of fast DNA sequencing (Min et al., 2011), scaffolds for tissue regeneration (Goenka et al., 2014; Geetha Bai et al., 2019; Maleki et al., 2020) and more (Allen et al., 2010; Krishna et al., 2013; Chabot et al., 2014; Kravets et al., 2015; Shehzad et al., 2016; Ahmad et al., 2018; Mohan et al., 2018). The successful combination of two or more components can generate composite materials, which show the individual properties of each component as well as completely new ones (Bottari et al., 2017). This is currently expanding the fields of application of existing materials and opening new avenues across different scientific fields, enhancing their technological progress. In literature, there is a vast amount of scientific articles concerning the methods to functionalize graphene and its derivatives (Georgakilas et al., 2012). To date, graphenes have been combined and functionalized with numerous categories of small or large organic molecules, metal atoms (Lawal, 2019), nanoparticles (Bottari et al., 2017), polymers (Georgakilas et al., 2012; Bottari et al., 2017), biopolymers (Sayyar et al., 2017; Ma et al., 2019; Bhasha et al., 2021), and a variety of other biomolecules (Li et al., 2016). Functionalization strategies vary according to the type of graphene and the nature of the component used to functionalize it. Numerous reviews categorize GBMs according to the methodologies employed for their manufacturing (Sayyar et al., 2017; Mohan et al., 2018; Ma et al., 2019; Al Faruque et al., 2021), the molecules to functionalize them (Bottari et al., 2017; Sayyar et al., 2017; Yu et al., 2020), the types of bonds between the components (Georgakilas et al., 2012), their architecture (Chabot et al., 2014; Sayyar et al., 2017), chemical and physical properties (Sayyar et al., 2017), applications (Ma et al., 2019; Dhinakaran et al., 2020) and more. For example, there are two main categories to classify GBMs based on the bonds between graphenes and the components with which they are functionalized. These are the functionalizations *via* covalent bonds, usually mediated by the use of cross-linker molecules, free radicals, dienophiles, chromophores and polymers, and non-covalent bondings such as π˗π, cation˗π, anion˗π, nonpolar gas˗π and H˗π interactions (Georgakilas et al., 2012). GBMs can also be categorized based on their architecture. Due to the nanoscopic dimensions of graphenes, it is possible to assemble 0D, 1D and 2D materials. These are often dispersed in liquid environments working as molecular probes (Sekhon et al., 2021) or assembled in monolayers for the fabrication of sensors (Garcia-Cortadella et al., 2021). However, the production of scalable 3D GBMs is possible under particular conditions and using specific components (Li et al., 2019; Bellucci and Tozzini, 2020). Among many approaches to fabricate 3D GBMs, the use of biomolecules is extremely popular due to numerous advantages linked to functionality, production time, costs, materials and sustainability. Moreover, considering the surface chemistry of biomolecules, GO is considered the most suitable graphene derivative to be combined with them due to its oxygen-containing groups. Therefore, it is important to discuss more specifically the types of biomolecules, as well as the currently available assembly methodologies to produce graphene oxide-based biomaterials (GOBBs).

### Graphene Oxide-Based Biomaterials

An extremely appealing alternative method for the fabrication of 3D GBMs is to employ biomolecules and biopolymers such as carbohydrates, lipids, aptamers, nucleic acids, small peptides, proteins, enzymes and viruses.

Nowadays biopolymers are very popular for the fabrication of GOBBs. This class of molecules is characterized by many repeated units called monomers, joined *via* covalent bonding to form long and large structures (Reddy et al., 2021). They can be classified based on their origin, type of monomers, production method, biodegradability and more (Reddy et al., 2021). For example, there are natural biopolymers such as collagen and its derivative gelatin which are protein-based and are generally obtained by the industries that process meats and fish (Gómez-Guillén et al., 2011). There are also cellulose, starch, amylose and many other polysaccharides obtained from plant sources (Singh et al., 2021). Alternatively to natural biopolymers, there are also synthetic biopolymers that are synthesised in the laboratory starting from natural sources or chemically modified versions of the ones available in nature (e.g., PCL, PLA, PLGA, PVA and others) (Reddy et al., 2021). As mentioned before, biopolymers are extremely popular in the field of GBMs, in particular for the production of GOBBs. These can be fabricated by mixing GO with natural or synthetic biopolymers, as well as a combination of both to provide specific properties to the final material (Ege et al., 2017; Sayyar et al., 2017; Aidun et al., 2019; Zhang et al., 2021). Although biopolymers are structurally simple molecules, they can covalently bind graphene and other derivatives *via* cross-linking agents or other physical methods (e.g., high pressure and temperature) (Sujan et al., 2021). Alternatively, GO can interact with biopolymers *via* weak intermolecular interaction in aqueous buffers. Non-covalent interactions can naturally occur or be favoured *via* specific modification of the individual components (Georgakilas et al., 2012; Li et al., 2016).

The assembly methods based on intermolecular interactions show several advantages compared with covalently bonded materials such as the possibility to perform reversible reactions in aqueous solutions at standard conditions of temperature, pressure and pH without altering the nature of the individual components (Jin et al., 2012; Pandit and De, 2020). Bio-functionalization is also advantageous as biomolecules can be produced or extracted on a large scale, using sustainable methods at relatively low costs. For example, recombinant proteins can be produced on large scale employing genetically engineered microorganisms (e.g., bacteria and yeasts) with a gene encoding for the protein of interest. Then, the microorganism can be grown in large fermenters while inducing the synthesis of the protein (Puetz and Wurm, 2019). Although the cost of recombinant proteins employed in medical applications can be extremely expensive due to higher quality requirements (10m-10bn$ Kg^−1^), the production cost of proteins employed in the industry can be as low as 10–100$ Kg^−1^ (Puetz and Wurm, 2019). Moreover, biomolecules are naturally biodegradable *via* the activity of a variety of enzymes produced by living organisms. Therefore, GOBBs are generally considered eco-friendly and biodegradable (Zhao et al., 2014; Aidun et al., 2019; Geetha Bai et al., 2019; Silva et al., 2021), excluding those made with non-biodegradable biopolymers (e.g., Bio-PET, Bio-PE and similar) or other synthetic and chemically modified biomolecules (Reddy et al., 2021). It is important to mention that the biodegradation of graphene and its derivatives is a currently popular topic (Chen et al., 2017). Graphenes have been found in the environment, raising health and environmental concerns among scientists and the public, due to their potential toxicity for living organisms (Chen et al., 2017). Numerous studies have shown that graphenes can be degraded by specific enzymes such as horseradish peroxidase (Li et al., 2014), myeloperoxidase (Kurapati et al., 2015; Kurapati et al., 2018; Kurapati et al., 2021), xanthine oxidase (Sureshbabu et al., 2015), eosinophil peroxidase (Andón et al., 2013) and more (Chen et al., 2017). Moreover, Peng et al. recently provided the first evidence of biodegradation of GO in the zebrafish gastrointestinal tract (Peng et al., 2020). Although the number of studies about graphenes biodegradation is rapidly growing, the current knowledge about it is limited, and further investigations are needed to elucidate the molecular mechanisms involved in these processes.

Apart from biopolymers which are extremely popular for the fabrication of GOBBs, other biomolecules such as nucleic acids (e.g., DNA and RNA), proteins (e.g., enzymes and antibodies) and entire viruses are of particular interest. Their monomers are nucleotides and amino acids, respectively, while viruses are larger particles, essentially comprised of a capsid made of proteins that surrounds a molecule of nucleic acid (Stephanopoulos, 2020). These biomolecules are characterized by high structural complexity, specific binding capability, catalytic activity and in some cases high stability in extreme conditions on temperature pressure and pH (Stephanopoulos, 2020; Ardini et al., 2021). DNA (deoxyribonucleic acid) is a polymer of nucleotides forming a double-helix of antiparallel strands. These, have a backbone of sugar and phosphate, and are connected through interactions between their nucleotide bases (adenine, thymine, cytosine and guanine). The biological role of DNA is to store the genetic information of the organism to which it belongs, within genes encoding for proteins (Madsen and Gothelf, 2019). Not only can DNA be produced on a large scale *via* bioprocesses, but it is also possible to generate highly specific sequences *via* chemical synthesis (Madsen and Gothelf, 2019). In the field of nanotechnology, DNA has been used for its ability to pair complementary sequences to form larger and more complex structures as in the case of DNA origami (Dey et al., 2021). Furthermore, this capability has also been employed to build molecular probes for the recognition of specific molecular targets *via* small DNA fragments (oligonucleotides of 13–25 nucleotides) (Madsen and Gothelf, 2019). Proteins are polymers of amino acids, naturally produced in living organisms by ribosomes during the process of translation (Hershey et al., 2012). The polymers of amino acids are subsequently processed by the molecular chaperones which assist their correct folding to ensure their functions. This process is fundamental as each protein covers one or multiple specific functions depending on its structure (Englander and Mayne, 2014). There are numerous functions in which proteins are involved within organisms, such as enzymatic activity (e.g., oxidoreductases, transferases, hydrolases, lyases, ligases, and isomerases), transport (e.g., haemoglobin and albumin), structure (e.g., actin, tubulin, keratin), defense (e.g., immunoglobulins) and more. Noticeably, both DNA sequences and proteins can be customized to carry specific modifications to accommodate specific needs for application in bio-nanotechnology and synthetic biology (Eckhart et al., 2020; Stephanopoulos, 2020; Hernandez-Garcia, 2021). These characteristics make these biomolecules extremely appealing and for some aspects, superior to biopolymers. These functional biomolecules are usually employed for the production of 2D GOBBs working as molecular probes for the transport or recognition of specific DNA sequences (Giuliodori et al., 2017; Wu et al., 2018) or to catalyse specific reactions (Hermanová et al., 2015; Zhou, 2021). Generally, these functionalized GO sheets are employed in solutions and cannot be scaled up into 3D materials. However, the possibility of manufacturing 3D materials capable of specific binding properties and catalytic activities due to embedded biomolecules is of high interest. The current literature about this class of composite materials is limited compared to the one of GOBBs assembled using more conventional biopolymers.

**FIGURE 2:**
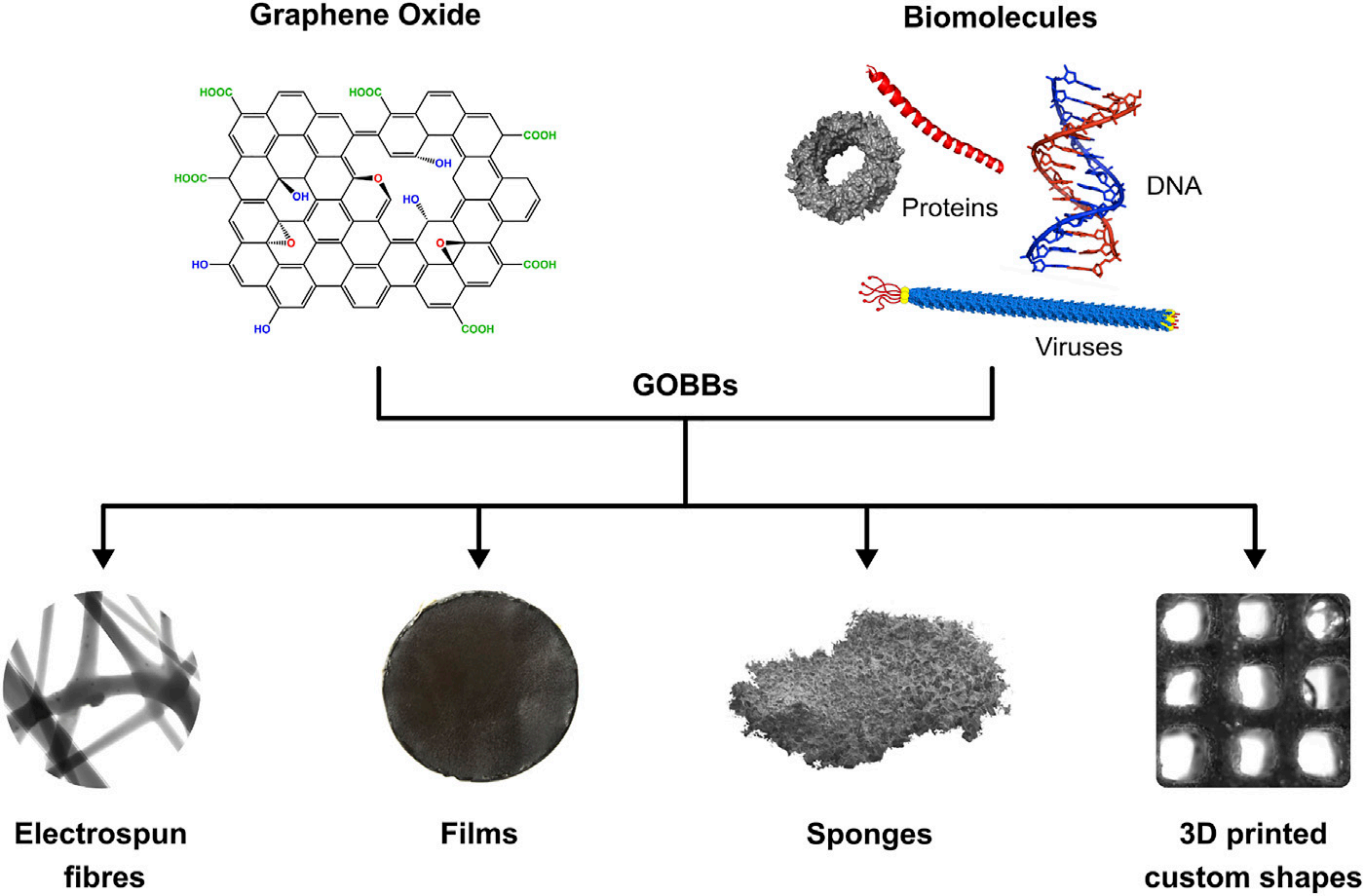
Schematic of GOBBs assembly GO and biomolecules can be combined to form GOBBs presenting different 3D structures such as fibres, films, sponges and 3D printed custom shapes.

The most common 3D structures currently produced using GOBBs include fibres, films, sponges and 3D printed custom shaped materials (Figure 2). Apart from fibres and 3D printed materials that require dedicated equipment, film- and sponge-like GOBBs are commonly made *via* solvent casting, salt leaching and freeze-drying methods (Sayyar et al., 2017). The resulting 3D GOBBs can be self-supporting (Xu et al., 2010; Ardini et al., 2016; Passaretti et al., 2019) and stretchable (Kim et al., 2020), as well as exhibiting low density (Passaretti et al., 2019) and extraordinary surface area (Passaretti et al., 2019), with remarkable mechanical properties (Xu et al., 2010), catalytic activity (Xu et al., 2019; Zhang et al., 2021) and low cytotoxicity (Coleman et al., 2017). This is particularly interesting for the fabrication of functional materials for applications in energy storage devices, sensors, catalysis, air and water filtering, as well as medicine (Shehzad et al., 2016; Bottari et al., 2017; Mohan et al., 2018; Maleki et al., 2020). These hierarchical assemblies attracted considerable attention and are expected to play a central role in a wide range of future technologies. Following, several examples of GOBBs fibres, films, sponges and 3D printed custom shapes are reported and discussed.

#### Fibre-Like GOBBs

**FIGURE 3:**
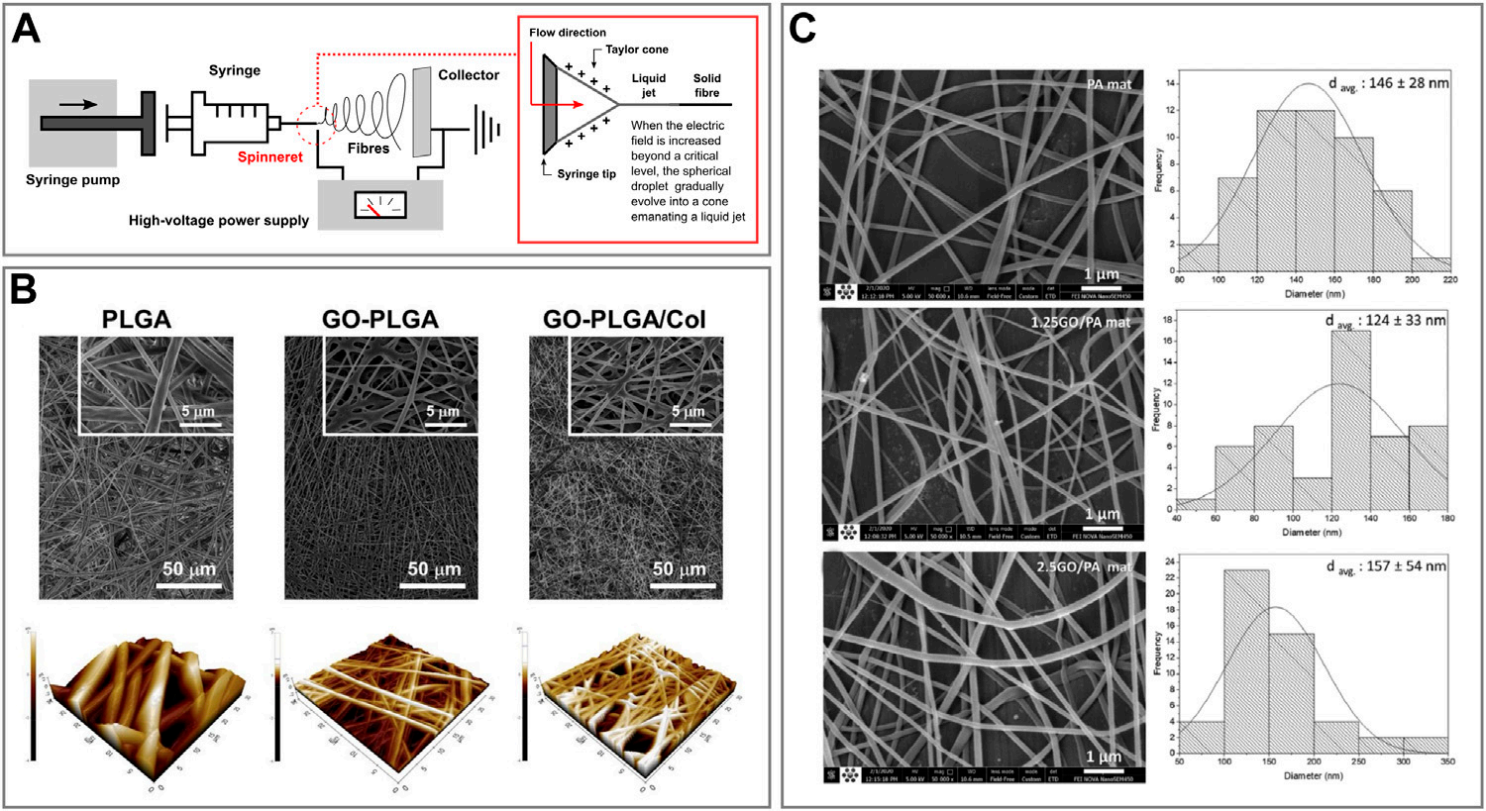
Fibre-like graphene oxide-based biomaterials **(A)** Schematic representation of an electrospinning setup and phenomenon of electrospinning. **(B)** FESEM (top) and AFM 3D rendered (bottom) images of PLGA, GO-PLGA and GO-PLGA-Col hybrid fibres. Reprinted with permission (Lee et al., 2014). **(C)** FESEM images of PA, 1.25GO-PA, 2.5GO-PA fibres (left) and histograms of their diameter distribution (right). Reprinted with permission (Bateni and Hashemi Motlagh, 2021).

Electrospinning has gained significant interest in recent years as it provides a versatile tool for the production of fibres with adjustable diameters ranging from nano to sub-micron scale, as well as matrices with uniform pore sizes (Al-Dhahebi et al., 2020). Moreover, it is considered the simplest and most economically viable method for the large-scale production of nanofibres (Wang et al., 2021). Essentially, electrospinning is a process that employs a strong electrical field to transform a polymer solution into fine filaments. A basic electrospinning setup consists of a syringe/container to pump the spinning fluid through a needle nozzle, a high-voltage power supply and an electrode collector. During the electrospinning process, the viscous spinning fluid is charged by the high voltage and drawn into a thin liquid jet by electrostatic forces. At the same time, solvent evaporation from the thin liquid jet turns it into solid fibres which are usually randomly deposited in the form of an entangled nanofibre mat, and collected *via* an electrode collector (Figure 3A) (Wang et al., 2021). Electrospinning has been successfully applied to produce fibres from a wide range of materials, including ceramics and metals, as well as graphene and its derivatives in combination with biopolymers and other biomolecules (Zhao et al., 2018a; Silvestri et al., 2019; Xue et al., 2019). Graphenes can be incorporated into electrospun fibres *via* two main approaches named pre-, and post-processing methods. The first method is characterized by the direct blending of the constituents, graphene included, and the *in situ* synthesis of the composite material. On the other hand, in the second method, fibres are coated with graphenes *via* physical dip-coating, ultrasonication, plasma treatment, wet chemical method and radiation treatment after being electrospun (Salavagione, 2018; Lawal, 2019; Al-Dhahebi et al., 2020).

Lee et al. assembled GO-decorated hybrid fibre sheets made of different combinations of poly (lactic-co-glycolic acid) (PLGA) and collagen (Col) *via* dual electrospinning (Lee et al., 2014). They produced GO-PLGA and GO-PLGA-Col hybrid fibres *via* combining the specific components in 1,1,1,3,3,3,-hexafluoro-2-propanol (HFIP) solvent. Subsequently, for the electrospinning procedure, the solutions were loaded into a syringe and pumped through a needle (21–25G) with a flow rate of 0.5 ml h^−1^. A 10 kV positive voltage was applied and there was a working distance of 11–12 cm between the needle tip and the collecting drum. In particular, GO-PLGA-Col fibres were collected on a rotating drum wrapped with aluminium foil while rotating at 20 rpm (Figure 3A). Finally, they compared the adhesion of human dermal fibroblasts (HDFs) from neonatal dermis onto the different hybrid fibres obtained. The results showed that HDFs could better adhere to GO-PLGA-Col fibres compared to the others, therefore, these could find potential applications as skin tissue engineering scaffolds (Lee et al., 2014). Similarly, Shin *et al.* produced hybrid fibre matrices composed of PLGA, collagen and GO (GO-PLGA-Col) *via* an electrospinning process (Shin et al., 2015). These hybrid matrices were comprised of randomly-oriented continuous fibres with a 3D non-woven porous structure. Moreover, the material was proved to be a promising biocompatible and biofunctional scaffold that can stimulate the differentiation of skeletal myoblasts while enhancing their attachment and proliferation (Shin et al., 2015).

On the other hand, Bateni et al. produced, characterized and compared several electrospun fibres using different combinations of polyethylene (PE) with polyamide (PA6) and GO (Bateni and Hashemi Motlagh, 2021). The electrospun solutions were prepared by mixing and sonicating the components in formic and acetic acids. Then, the resulting solutions were electrospun at room temperature, 20 kV at a rate of 0.3 ml h^−1^, and collected *via* a rotating collector placed at a distance of 25 cm while spinning at 1,500 rpm (Figure 3B). Finally, Hajebi et al. developed a GO-polyamide-polypyrrole (GO-PA-PPy) nanofibre fabricated *via* electrospinning technique and successfully tested it for solid-phase microextraction of methamphetamine from urine samples (Hajebi et al., 2020).

#### Film-Like GOBBs

Film-like GOBBs are usually produced through conventional casting methods that do not require sophisticated equipment. Commonly, GO is dispersed in solution together with the biomolecule of interest and the solution or hydrogel obtained is deposited on a support allowing the solvent to naturally evaporate or employing a drying procedure. Alternatively, the hydrogel can be deposited on a filter and the solvent removed by applying a vacuum with a rotary pump (Sayyar et al., 2017).

**FIGURE 4:**
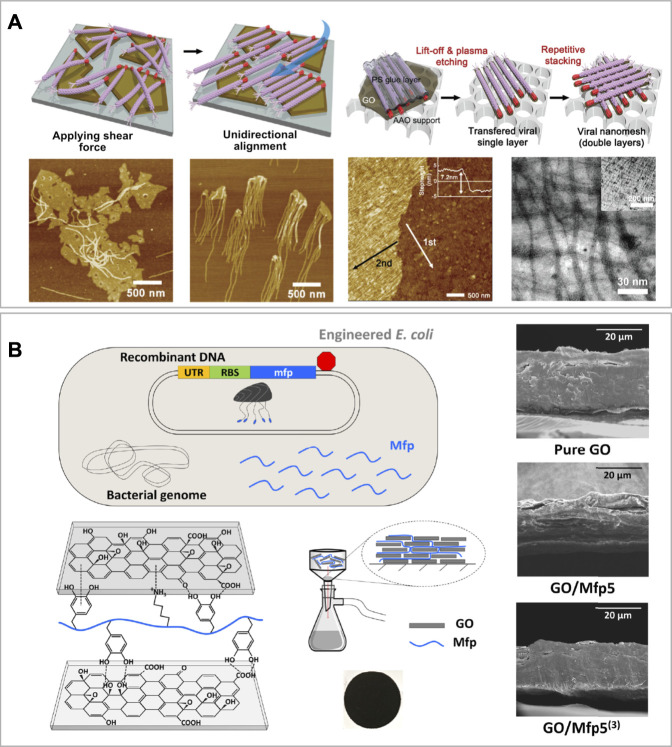
Film-like graphene oxide-based biomaterials **(A)** Schematic illustration of the unaligned vs. aligned M13 viruses bond onto the edges of GO sheets via the GOBP (red) and below, the corresponding AFM images of the samples. At the right-hand side, the schematic procedure of viral layer transfer and stacking, with corresponding AFM and TEM images of the bilayered viral nanomesh structure. Reprinted with permission (Lee et al., 2014). **(B)** Schematic of genetically engineered *E. coli* to overexpress Mfp. On the left-bottom corner, the schematic of DOPA and lysine residues on recombinant Mfp interacting with oxygen-functional groups of GO nanosheets and experimental set-up to assemble the GO/Mfp mixture onto a PES support membrane, resulting in a thin film composite (showed below). On the right-hand side SEM cross-section images of the resulting GO/Mfp composite material films made of pure GO, GO/Mfp5 and GO/Mfp5^(3)^. Reprinted with permission (Kim et al., 2020).

Lee et al. designed and realized a highly selective ultrathin membrane having a nanomesh structure made of unidirectionally aligned M13 filamentous viruses attached to GO nanosheets (Figure 4A) (Lee et al., 2014). GO was prepared using Hummers’ method, while M13 was genetically engineered to expose on its PIII proteins a GO binding peptide (GOBP). The GOBP sequence was −CHKKPSKSC−, where C, H, K, P, and S refer to the amino acids cysteine, histidine, lysine, proline, and serine, respectively. The self-assembly of GO and M13 was mediated by hydrogen bondings between the oxygen-containing groups present on the GO edges and the GOBP in a pH range between 3.0–7.0. Moreover, M13 was subsequently unidirectionally oriented applying a shear force (Figure 4A). To do so, Lee et al. considered that the PVIII protein of M13, which represents the majority of the virus body, was negatively charged at pH > 4.8. At these conditions, M13 can be weakly repelled by the abundant number of negatively charged groups present on the GO (Passaretti et al., 2020a). Subsequently, they identified that the optimal condition to assemble and orient M13 onto GO, as well as remove the majority of impurities, was at pH 6.5. Once formed unidirectionally aligned layers of GO-M13, those were thermally cross-linked, overlapped and perpendicularly oriented to obtain a nanomesh structure of 10–30 nm thickness (∼7–8 nm each layer). Finally, the resulting ultrathin membrane was tested for filtering applications (Lee et al., 2014).

Kim et al. synthesized a film composite material using GO and mussel foot proteins (Mfp) of the saltwater mussel *Mytilus galloprovincialis* (Kim et al., 2020). They firstly engineered an *Escherichia coli* strain to express the Mfp, and subsequently, performed a post-translational modification step to add residues of the non-canonical aminoacid 3,4-dihydroxyphenylalanine (DOPA) onto it (Figure 4B). For this study, they used a specific Mfp, the Mfp5 and a synthetic protein consisting of three consecutive repeats of Mfp5 named Mfp5^(3)^ (Kim et al., 2018). Therefore, they synthesized GO *via* a modified Hummers’ method, and then, they assembled the film-like composite through an aqueous-based green synthesis strategy. This method consisted in mixing GO of 0.1 mg ml^−1^ with 1 mg of either Mfp5 or Mfp5^(3)^ in 0.5% acetic acid. The mixture was sonicated and poured onto a PES membrane support and the solution was passed through *via* vacuum filtration. Finally, the film was soft-baked at 37°C for 1 h. This facile method was used to produce GO-Mfp films with remarkable mechanical properties such as high-tensile strength (134–158 MPa), stretchability (∼26% elongation) and high-toughness (20–24 MJ m^−3^). The authors also suggested that if further optimized, this material could find application in bioelectronic as a wearable device to convert physical resistance into electrical signals (Kim et al., 2020).

Similarly to these two works, there are other film-like composites reported in the literature, assembled using GO and other proteins. Vural et al. successfully synthesized and characterized a hybrid film-like material consisting of GO and an engineered version of the squid ring teeth (SRT) proteins (Vural et al., 2017). Moreover, (Xie et al., 2018) and (Zhao et al., 2018b) employed silk fibroin combined with GO and rGO, respectively, to form film-like GOBBs.

#### Sponge-Like GOBBs

Sponge-like GOBBs are usually assembled in a similar way to film-like materials. GO and the chosen biomolecule are dispersed in solution to form a hydrogel that is subsequently subjected to freeze-drying (Sayyar et al., 2017). Therefore, the hydrogel is cooled down to its frozen state allowing the solvent to form ice crystals, which are then sublimed producing pores within the material.

Xu et al. developed a novel and facile 3D self-assembly method to prepare a composite hydrogel/aerogel using 20–30 bp fish sperm double-stranded DNA (dsDNA) fragments and GO. They mixed in an aqueous solution GO (6 mg ml^−1^) and dsDNA (10 mg ml^−1^) with a ratio of 1:1 by volume. Subsequently, they heat the homogeneous mixture at 90°C for 5 min allowing the DNA to unwound to ssDNA and interconnect adjacent GO sheets *via* non-covalent interactions (Xu et al., 2010). The final material was successfully tested as a dye adsorber using sefranine O and its self-healing properties were also shown.

**FIGURE 5:**
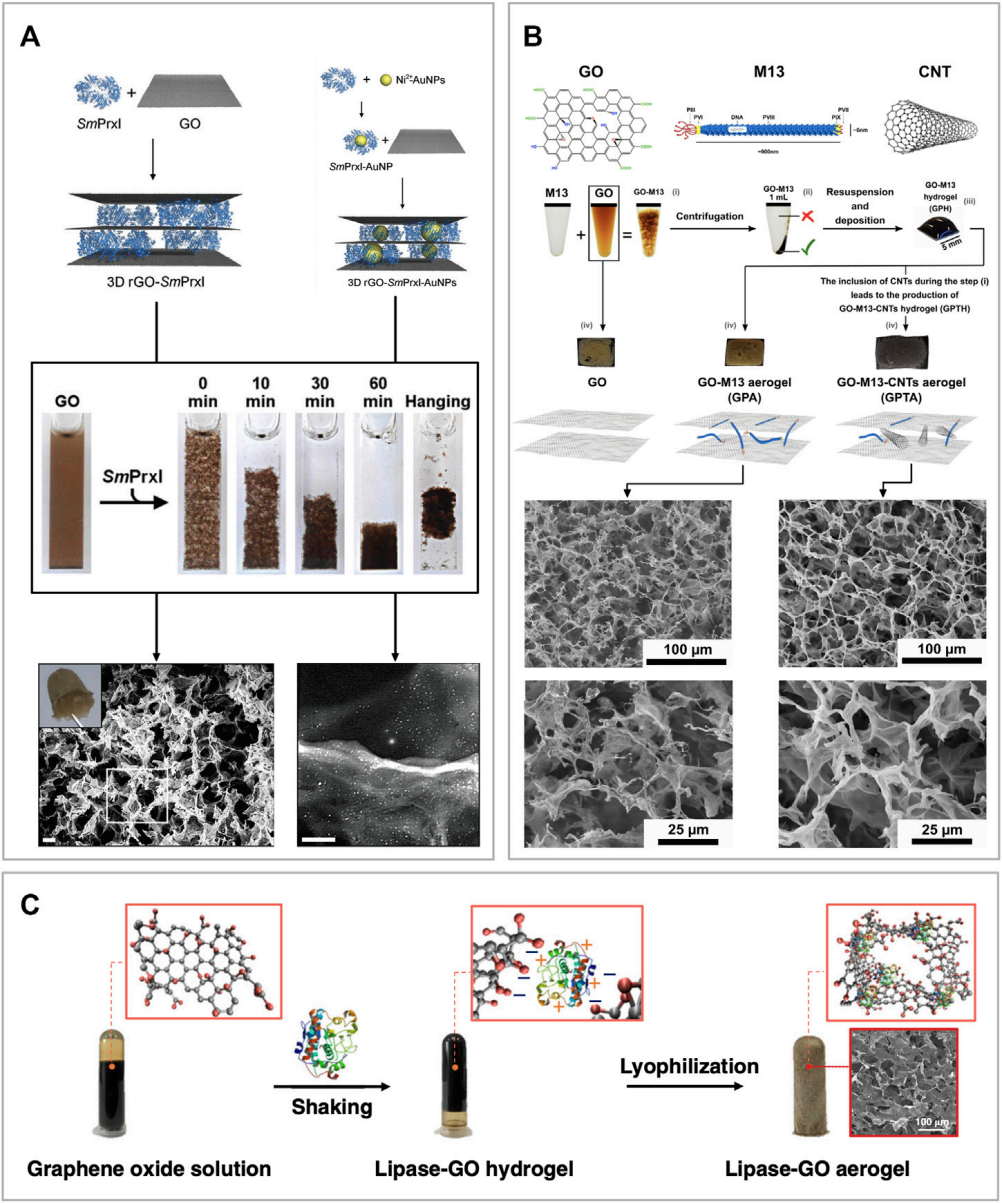
Sponge-like graphene oxide-based biomaterials **(A)** Schematic of the self-assembly mechanism where *Sm*PrxI rings adhere flat over the surface of GO (top left corner). The same approach was employed to self-assemble the same composite but functionalized with gold nanoparticles bonded to the *Sm*PrxI (top right corner). The reaction is also shown in the picture where it is possible to see the GO in suspension, starting to assemble after adding the *Sm*PrxI, and forming a hydrogel. At the bottom of the figure, the corresponding SEM images of the two composite materials after being freeze-dried to form an aerogel. Moreover, a picture of the final aerogel is shown in the top left corner of the GO-*Sm*PrxI SEM image. Reprinted with permission (Ardini et al., 2016). **(B)** Schematics of GO, M13 bacteriophage and CNT, followed by an overview of the self-assembly process of *GraPhage13* aerogels. The process is divided into four steps: i) assembly, ii) precipitation, iii) deposition and iv) drying. Images of the aerogels of GO, GO-M13 and GO-M13-CNT, as well as secondary electron SEM images of the samples at two different magnifications. Reprinted with permission (Sun et al., 2020). **(C)** Schematic of the one-step co-gelation and lyophilization after mixing GO and CALB solutions to prepare GO-CALB functional aerogel. Reprinted with permission (Xu et al., 2019).

Ardini et al. developed a 3D free-standing porous material combining GO and a ring-like protein called peroxiredoxin I from *Schistosoma mansoni* (*Sm*PrxI) (Figure 5A). They showed that this protein can self-assemble with GO sheets dispersed in an aqueous solution at neutral pH, interacting *via* weak intermolecular interactions. Moreover, due to the presence of cysteine residues, *Sm*PrxI is also able to simultaneously reduce GO (Ardini et al., 2016; Ardini et al., 2021). Furthermore, due to the presence of several His-tags exposed in its lumen, *Sm*PrxI can bind gold nanoparticles functionalized with nitrilotriacetic acid (NTA) in presence of nickel, or even promote the *in situ* growth of palladium nanoparticles. The GO-*Sm*PrxI is prepared in an aqueous solution, where the components self-assemble and separate from the supernatant. The latter is promptly removed and the remaining hydrogel is subject to freeze-drying. The resulting material is a brownish free-standing porous aerogel, extremely light and delicate. Due to its properties, flexibility and scalability, this material can find applications in the fields of chemical catalysis, optoelectronics, environmental recovery and bio-scaffold generation (Ardini et al., 2016).

Alternatively, Passaretti et al. combined the filamentous bacteriophage M13 with GO to assemble a similar porous structure named *GraPhage13* (Figure 5B) (Passaretti et al., 2019; Passaretti et al., 2020a; Passaretti et al., 2020b). They showed that M13 and GO can reversibly self-assemble in an aqueous solution at specific pH values. Once the two components form the GO-M13 hydrogel, this can be freeze-dried to obtain a sponge-like material characterized by high porosity and extremely low weight. In addition to this work, Sun et al. also demonstrated that it is possible to include carbon nanotubes (CNTs) during the assembly process, producing a structurally similar sponge (Figure 5B) (Sun et al., 2020).

Xu et al. were the first to develop a facile method to engineer a water-like microenvironment for gas-phase enzymatic reactions by embedding the *Candida antarctica* lipase B (CALB) in a hydrophilic, hydroxyl-rich GO aerogel matrix (Figure 5C). This enzyme is a hydrolase that catalyses the hydrolysis of triglycerides into fatty acids and glycerol. To assemble the GO-CALB composite material, they firstly mix the two components in solutions at pH 3.3 to form a hydrogel which was subsequently subject to freeze-drying at −40°C for 50 h to form an aerogel. Finally, the material was characterized and tested showing that the lipase immobilized in the GO-CALB exhibits a 5 to 10-fold increase in apparent activity than the lyophilized lipase powder used as control. Moreover, the enzymatic activity was maintained for more than 500 h, making this material promising for applications in gas-phase enzymatic catalysis (Xu et al., 2019).

#### 3D-Printed GOBBs

3D printing has revolutionized the way of designing and manufacturing complex structures, favouring the production of functional materials with superior properties such as mechanical, electrical, thermal and more. Therefore, considering the appealing properties of graphenes, 3D printing has been also widely employed for the fabrication of numerous GBMs and GOBBs (Silva et al., 2021). Generally, 3D printing methods are essentially based on modelling software to generate a 3D model, then converted in an STL file format. Subsequently, the STL file data are converted into a G-code file containing 2D layers information corresponding to the sliced version of the 3D model. Finally, the 3D model is printed in a layer-by-layer manner by a printing apparatus (Ambrosi and Pumera, 2016). 3D printing can be grouped into four main categories depending on which way the 2D layers are deposited, and these include the photopolymerization, extrusion, powder-based and lamination methods (Ambrosi and Pumera, 2016). All these categories include specific methods suitable for specific applications. In particular, extrusion methods like direct ink writing (DIW) (Yun et al., 2019) and fused deposition modelling (FDM) (Cardoso et al., 2020), as well as photopolymerization and powder-based methods like stereolithography (SLA) (Palaganas et al., 2019) and selective laser sintering (SLS) (Shuai et al., 2015), are typical techniques for the fabrication of GBMs and GOBBs (Silva et al., 2021).

**FIGURE 6:**
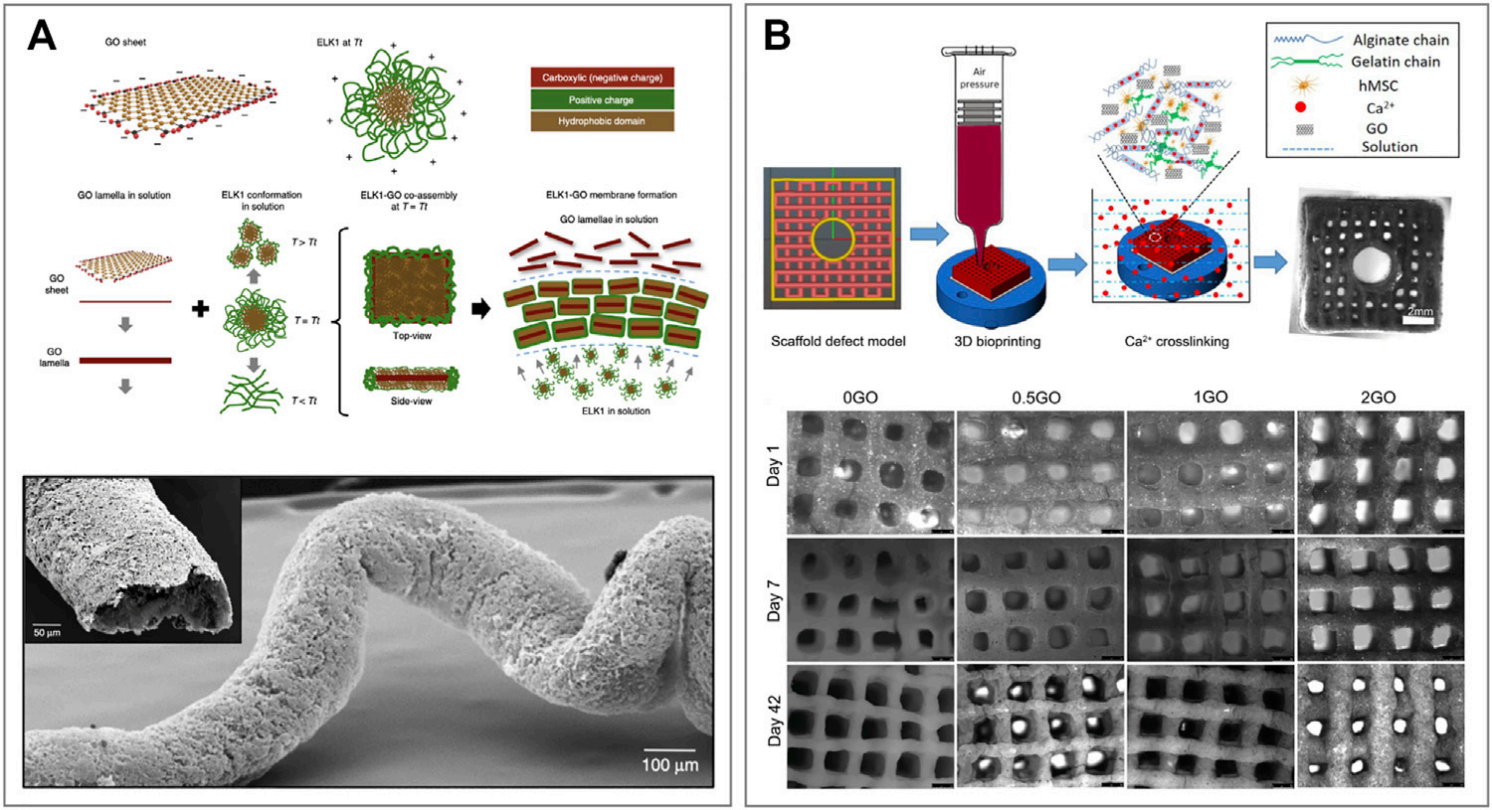
3D printed graphene oxide-based biomaterials **(A)** Schematic representation of a GO sheet and ELK1 at its transition temperature (*Tt* = 30°C) indicating the charged (red and green) and hydrophobic (brown) parts of both components. The proposed assembly mechanism showing the conformation of GO and ELK1 before and after co-assembly. Images of the 3D printed GO-ELK1 material for the fabrication of fluidic devices with tubular structure. Reprinted with permission (Wu et al., 2020). **(B)** Schematic illustration of 3D bioprinting pipeline and the 3D cell-laden defect scaffolds (top). Images of the 3D cell-laden defect scaffolds with different GO contents and cultured in the osteogenic media for 1, 7 and 42 days (bottom). Reprinted with permission (Zhang et al., 2021).

Wu et al. developed a co-assembling system to generate hierarchically organized materials with high stability *via* a diffusion-reaction process and disorder-to-order transitions (Figure 6A) (Wu et al., 2020). To do so, they employed GO and different elastin-like recombinamers (ELRs) proteins named ELK0, one and three due to their, respectively, increasing amount of positively charged lysine residues. ELRs are elastin-like polypeptides based on the natural elastin motif −VPGXG−, where V, P and G refer to the amino acids valine, proline and glycine, and X could be any amino acid apart from proline. ERLs were synthesized *via* an *E. coli* recombinant expression system (Urry et al., 1974). These biomolecules are capable of temperature-dependent reversible-phase transitions, which makes them an appealing component to produce biocompatible materials (Girotti et al., 2004). In this study, ELK1 was chosen among the other ELRs due to its easily accessible transition temperature (*Tt*) of 30°C and its ability to establish cooperative interactions *via* its charged and hydrophobic segments with the anionic edge and hydrophobic surface of the GO, respectively (Figure 6A). Similarly to other GOBBs, the assembly strategy is performed in an aqueous solution where the two components are mixed, reacting in a very short time. GO-ELK1 was subsequently loaded onto a 3D printer to produce custom tubular shapes. Finally, the resulting GO-ELK1 materials have been extensively characterized and tested for applications as functional microfluidic systems, tissue-engineered scaffolds and organ-on-a-chip devices.

On the other hand, Zhang et al. developed a GO-alginate-gelatin composite bio-ink to form 3D bone-mimicking scaffolds using a 3D bioprinting technique (Zhang et al., 2021). To do so, GO solutions of 0, 0.5, 1 and 2 mg ml^−1^ were combined with alginate and gelatin in glycerol-PBS aqueous buffer. The resulting four different GO-alginate-gelatin ink solutions were named 0, 0.5, 1 and 2GO, respectively (Figure 6B) (Zhang et al., 2019). The printing process was performed at a temperature range between 10–15°C with the addition of polyethylene to allow printability. Moreover, the printing speed was set at 2 mm s^−1^ through a 27 G nozzle at an air pressure of 50 kPa. Immediately after printing, the scaffolds were cross-linked with CaCl_2_ solution to maintain structural integrity for long-term stability, and the excess of CaCl_2_ was washed with cell culture media. Once the optimum printing conditions for the GO-alginate-gelatin bio-ink were established, human mesenchymal stem cells (hMSCs) were included in the formulation, producing a (hMSCs)-laden bio-ink. The (hMSCs)-laden scaffolds were printed with the same specifics and cultured in osteogenic media for different periods of time (Figure 6B). Finally, the properties of hMSCs-GO-alginate-gelatin scaffolds were characterized, and cell viability and proliferation assays were performed. The results showed that GO composite bio-inks, and in particular 1GO bio-ink, have better printability and scaffold fidelity than the other formulations tested. Moreover, they showed a better cell proliferation, osteogenic differentiation and extracellular matrix (ECM) mineralization than controls, demonstrating great potential for 3D bioprinting of bone tissue models and tissue engineering applications (Zhang et al., 2021). Similar works have been conducted by Cheng *et al.* for the construction of a cartilage matrix (Cheng et al., 2020) as well as Belaid *et al.* for bone formation and tissue regeneration (Belaid et al., 2020).

## Discussion

Biomolecules can be used to assemble fibres, films, sponges and custom shaped GOBBs for numerous applications. This is possible *via* conventional and simple approaches such as solvent casting and freeze-drying, as well as employing more sophisticated approaches requiring specific instruments like electrospinning and 3D printing.

The possibility to assemble GO in solution in a short time, avoiding costly equipment and polluting reagents makes this approach extremely appealing for the production of GOBBs. Furthermore, the possibility to produce biomolecules on large scale favours the scalability and mass production of this class of graphene-based biomaterials. However, the eco-compatibility and sustainability of GOBBs cannot be based only on the fact that their components can be naturally degraded in the environment, but it is also important to employ sustainable methods for their production, disposal and recycling. This includes sustainable approaches to produce both graphenes and biomolecules, as well as the methodologies and types of equipment employed to assemble GOBBs. Moreover, the confirmed presence of graphenes in the environment highlights the fact that although there are biological activities that can naturally degrade them, those might not be sufficient to avoid the accumulation of graphenes and GMBs in the environment, allowing them to become a toxic pollutant for living organisms.

Due to their natural compatibility, there are many strategies to combine biomolecules and GO in hybrid systems to produce functional GOBBs. For example, the employment of DNA for the production of GOBBs could be expanded by testing the selective assembly of GO nanosheets *via* engineered DNA sequences, combined with the activity of specific enzymes or antibodies to catalyse specific reactions or bind specific targets. Moreover, due to their size, viruses and bacteriophages like M13 can be genetically engineered or chemically modified to expose on their surface specific proteins or chemical groups while favouring the self-assembly of GO into functional hydrogel or aerogel. Therefore, considering the numerous biomolecules available as building blocks and the number of compatible modifications (both, chemical and genetic), the amount of potential GOBBs deriving from them is virtually uncountable.

Although biomolecules are naturally capable to self-assemble *via* extremely specific and sophisticated mechanisms (e.g., the specific assembly of protein complexes and enzymes activity), it remains very difficult to create highly ordered structures when combined with GO. This is due to the variable GO sheets size as well as the heterogeneous abundance and distribution of the oxygen-containing groups and defects on the surface and edges of GO. Therefore, the production of graphenes with more homogeneous characteristics is crucial for the development of GOBBs showing highly-ordered hierarchical nanostructures. Other disadvantages of the bio-functionalization of GO often include the structural fragility towards aqueous solvents and mechanical fragility due to the weak and reversible interactions between these components. Moreover, biomolecules tend to denature at extreme levels of pH and temperature, making GOBBs not suitable for applications in these conditions.

Notably, although the materials found in the literature have been extensively characterized and sometimes even tested for specific applications, they have not often been systematically compared with structurally similar graphene-based materials synthesized *via* more conventional chemical or physical methods. It is important to standardise the characterization methods of GOBBs as well as to provide information about the performance of specific materials and compare them to potential competing materials. Another important aspect that emerged in the literature analysis of GOBBs is that the design, manufacturing and characterization of these materials are not always followed-up by further work where the materials are optimized and tested in real applications. It is therefore important to try not only to assemble and characterize but also to test the efficiency, scalability, performance and sustainability of novel GOBBs in real applications.

## Conclusion and Outlooks

In this review, GOBBs assembled with biopolymers and other biomolecules such as nucleic acids and proteins are discussed. It is important to point out that these biomolecules are characterized by much more complex structure and chemistry than the group of other biopolymers mentioned above. These biomolecules are extremely appealing components to use for bio-functionalization due to their capability to mediate the self-assembly of graphenes and act as a platform for interactions with other components while showing catalytic activity or high selectivity to specific targets. However, bio-functionalization is still a considerable challenge for the fabrication of novel functional GOBBs. For example, protein functions are linked to their structural conformations and, therefore, their use is limited by their specific denaturing conditions of temperature, pressure and pH. More biomolecules need to be identified as potential candidates for the production of GOBBs based on their structural or catalytic properties. Moreover, *ad hoc* modifications need to be developed to expand the conditions where these biomolecules can be employed. Although the works carried out with biopolymers seem to be prevalent, the use of more complex biomolecules is certainly an alternative to be considered and experimented more in-depth. Therefore, it is crucial to design, produce and characterize novel 3D functional GOBBs with similar, if not superior, characteristics and performances compared to other GBMs while producing them through more sustainable methods.

## Glossary

0D: zero-dimensional
1D: one-Dimensional
2D: two dimensional
3D: three-dimensional
AFM: atomic force microscopy
Bio-PE: bio-polyethylene
Bio-PET: bio-polyethylene terephthalate
CALB: candida antarctica lipase B
CNT: carbon nanotube
Col: collagen
CVD: chemical vapour deposition
DIW: direct ink writing
DOPA: 3,4-dihydroxyphenylalanine
ECM: extracellular matrix
ELR: elastin-like recombinamers
FDM: fused deposition modelling
FESEM: field emission scanning electron microscopy
GBM: graphene-based material
GNR: graphene nanoribbon
GO: graphene oxide
GOBB: graphene oxide-based biomaterial
GOBP: graphene oxide binding peptide
GQD: graphene quantum dot
HFIP: 1,1,1,3,3,3,-hexafluoro-2-propanol
hMSC: human mesenchymal stem cells
LPE: liquid-phase exfoliation
NTA: nitrilotriacetic acid
PA: polyamide
PCL: polycaprolactone
PLA: poly(lactic acid)
PLGA: poly lactic-co-glycolic acid
PPy: polypyrrole
PVA: polyvinyl alcohol
rGO: reduced graphene oxide
SEM: scanning electron microscopy
SLA: stereolithography
SLS: selective laser sintering
*Sm*PxrI: Schistosoma mansoni peroxiredoxin I
SRT: squid ring teeth proteins
Tt: transition temperature
UV: ultraviolet
